# Interpretable machine learning identifies a clinically inferred inflammasome-associated inflammatory injury phenotype in children with adenovirus pneumonia

**DOI:** 10.3389/fcimb.2026.1902566

**Published:** 2026-07-17

**Authors:** Jie Li, Yabin Wu, Yang Huang, Wenhua Deng

**Affiliations:** Department of Pediatric Respiratory, Maternal and Child Health Hospital of Hubei Province, Wuhan, China

**Keywords:** adenovirus pneumonia, children, inflammasome, inflammatory injury, machine learning, severity discrimination, SHAP, temporal validation

## Abstract

**Background:**

Severe adenovirus pneumonia in children is characterized by persistent fever, systemic inflammation, and extrapulmonary tissue injury. Although adenovirus can activate inflammasome pathways, direct inflammasome biomarkers are not routinely available in pediatric practice. This study developed an interpretable machine-learning framework for retrospective severity discrimination and inflammatory phenotype stratification.

**Methods:**

We retrospectively analyzed 82 hospitalized children with adenovirus pneumonia, including 32 mild and 50 severe cases. Demographic, clinical, and laboratory variables were extracted from medical records. Laboratory variables were obtained from the first available blood tests within 24 hours after admission, whereas fever duration reflected the total febrile course. A surrogate Inflammasome-associated Inflammatory Injury Index (IAI) was calculated as the mean z-score of lactate dehydrogenase, C-reactive protein, aspartate aminotransferase, neutrophil-to-lymphocyte ratio, and maximal temperature. Logistic regression, support vector machine with radial basis function kernel, and random forest classifiers were evaluated using repeated stratified 5-fold cross-validation. SHAP values and unsupervised clustering were used for model interpretation and phenotype discovery. A small post-2019 cohort was used for exploratory single-center temporal validation.

**Results:**

Severe cases were younger and had longer fever duration, higher maximal temperature, lower hemoglobin, and higher alanine aminotransferase, aspartate aminotransferase, lactate dehydrogenase, and creatine kinase-MB levels than mild cases. IAI was significantly higher in severe disease. Random forest showed the best internal performance, with an AUC of 0.965 ± 0.031, PR-AUC of 0.980 ± 0.018, accuracy of 0.890, and F1-score of 0.913. Five-fold out-of-fold analysis yielded an AUC of 0.966 and a Brier score of 0.088. SHAP identified fever duration and IAI as the two leading contributors. Clustering identified an inflammatory injury-high phenotype comprising 11 patients, all with severe pneumonia. In exploratory temporal validation, the frozen random forest model achieved an AUC of 0.840 in 39 post-2019 cases.

**Conclusion:**

Routine clinical variables can support interpretable severity discrimination and phenotype discovery in pediatric adenovirus pneumonia. The inflammatory injury-high phenotype suggests a clinically detectable systemic inflammatory injury pattern biologically compatible with inflammasome-related inflammation. Because IAI is a surrogate clinical index rather than a direct molecular measure of inflammasome activation, prospective multicenter studies incorporating direct inflammasome biomarkers are required for biological validation.

## 1 Introduction

Human adenoviruses (HAdVs) are non-enveloped double-stranded DNA viruses that can cause a broad spectrum of respiratory, ocular, gastrointestinal, and systemic infections (Lynch and Kajon, 2016). In children, HAdV pneumonia represents an important cause of community-acquired pneumonia and may progress rapidly to severe pneumonia, respiratory failure, acute respiratory distress syndrome, and extrapulmonary organ injury (Lu et al., 2013; Wang et al., 2024). Timely recognition of children with severe disease is therefore clinically important, particularly because specific antiviral options remain limited and current management largely depends on supportive care, timely escalation of respiratory support, immunomodulatory treatment in selected cases, and management of complications (Wu et al., 2020; Zhang et al., 2023).

Accumulating experimental evidence indicates that inflammasome activation is involved in the host inflammatory response to adenovirus infection. Adenovirus membrane penetration has been shown to activate the NLRP3 inflammasome through endosomal or lysosomal perturbation and cathepsin B-associated signaling (Barlan et al., 2011). More recently, the HAdV-7 L4 100-kDa protein was reported to interact with NLRP3 and promote inflammasome assembly, providing a virus-specific molecular link between adenovirus infection and inflammasome activation (Duan et al., 2024). In parallel, HAdV-associated pulmonary inflammatory damage has been associated with inflammasome activation and macrophage pyroptosis (Li et al., 2023). These observations are consistent with the broader concept that viral infection can trigger inflammasome priming and activation, inflammatory caspase signaling, interleukin-1β/interleukin-18 maturation, gasdermin-mediated pyroptosis, and downstream inflammatory tissue injury. More broadly, inflammasomes and gasdermin-mediated pyroptosis are increasingly recognized as important components of antiviral immunity and virus-induced inflammatory tissue injury (Martinon et al., 2009; Schroder and Tschopp, 2010; Kelley et al., 2019; Swanson et al., 2019; Kuriakose and Kanneganti, 2023). These findings are particularly relevant to severe pediatric adenovirus pneumonia, which is frequently characterized by persistent fever, systemic inflammatory response, and multi-organ involvement. More recent reviews have further emphasized that NLRP3 inflammasome activation is regulated by complex priming, licensing, post-translational modification, assembly, and spatial regulatory networks, reinforcing the biological plausibility of inflammasome involvement in virus-induced inflammatory tissue injury (Paik et al., 2025).

Despite these mechanistic advances, direct measurement of inflammasome-related molecules, including NLRP3, caspase-1, IL-1β, IL-18, and gasdermin D, is not routinely available in most pediatric clinical settings. This gap limits the direct translation of inflammasome biology into bedside risk stratification. However, routine clinical and laboratory variables may capture downstream consequences of systemic inflammatory injury. For example, lactate dehydrogenase (LDH) reflects cellular injury and lytic damage, C-reactive protein (CRP) reflects systemic acute-phase inflammation, aspartate aminotransferase (AST) may indicate tissue or extrapulmonary injury, the neutrophil-to-lymphocyte ratio (NLR) reflects innate immune predominance and lymphocyte suppression, and body temperature reflects pyrogenic inflammatory signaling (Gabay and Kushner, 1999; Dinarello, 2009; Schroder and Tschopp, 2010; Swanson et al., 2019). Although these markers are not specific molecular readouts of inflammasome activation, their integrated pattern may provide a clinically accessible approximation of systemic inflammatory and tissue-injury burden that is biologically compatible with inflammasome-related inflammation.

Traditional clinical studies have identified several risk factors associated with severe adenovirus pneumonia, including prolonged fever, young age, respiratory distress, and inflammatory or tissue-injury biomarkers (Fu et al., 2019; Wang et al., 2023). Nevertheless, severe viral pneumonia is a heterogeneous syndrome driven by complex interactions among viral factors, host immune response, systemic inflammation, and organ injury. Conventional regression models may not fully capture nonlinear relationships among these variables. Machine-learning methods offer a flexible framework for integrating multidimensional clinical data and may improve severity discrimination in small but information-rich clinical cohorts (Cortes and Vapnik, 1995; Breiman, 2001). Importantly, interpretable approaches such as SHapley Additive exPlanations (SHAP) can provide feature-level explanations, allowing model predictions to be linked back to clinically meaningful biological patterns rather than functioning as purely black-box outputs (Lundberg and Lee, 2017; Lundberg et al., 2020). However, feature importance derived from interpretable machine learning should be understood as model-based association rather than evidence of causality.

**Figure 1:**
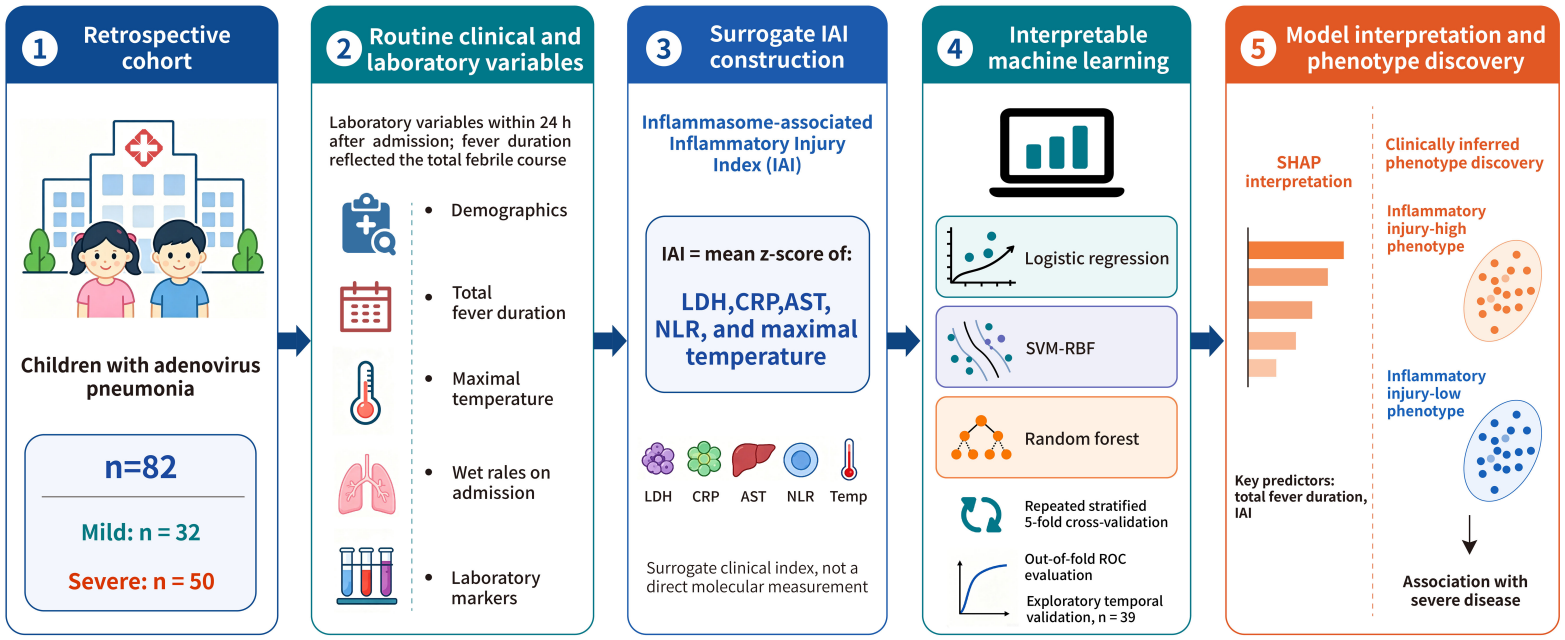
Overall study workflow for interpretable machine learning and clinically inferred inflammatory injury phenotype discovery in pediatric adenovirus pneumonia. This retrospective study included 82 children with adenovirus pneumonia, including 32 mild and 50 severe cases. Routine clinical and laboratory variables were extracted from medical records. Laboratory variables were obtained from the first available blood tests within 24 hours after admission, whereas fever duration reflected the total febrile course. A surrogate Inflammasome-associated Inflammatory Injury Index (IAI) was constructed as the mean z-score of lactate dehydrogenase (LDH), C-reactive protein (CRP), aspartate aminotransferase (AST), neutrophil-to-lymphocyte ratio (NLR), and maximal temperature. Three interpretable machine learning models, including logistic regression, support vector machine with radial basis function kernel (SVM-RBF), and random forest, were developed and evaluated using repeated stratified 5-fold cross-validation and out-of-fold receiver operating characteristic (ROC) analysis. An exploratory temporal validation was additionally performed in a post-2019 cohort (n = 39). Model interpretation was conducted using Shapley additive explanations (SHAP), and phenotype discovery identified a clinically inferred inflammatory injury-high phenotype and an inflammatory injury-low phenotype. Fever duration and IAI were the leading contributors to model prediction, and the inflammatory injury-high phenotype was associated with severe disease.

In this study, we retrospectively analyzed 82 hospitalized children with adenovirus pneumonia and developed an interpretable machine-learning framework for clinical severity discrimination and inflammatory phenotype stratification. We constructed a surrogate inflammasome-associated inflammatory injury index, termed the Inflammasome-associated Inflammatory Injury Index (IAI), using routine fever, inflammation, and tissue-injury markers. We then compared logistic regression, support vector machine with radial basis function kernel, and random forest classifiers, interpreted the best-performing model using SHAP, and performed unsupervised clustering to identify inflammatory injury phenotypes. Because fever duration reflected the total febrile course rather than a variable strictly available at initial presentation, the present framework was positioned as a retrospective severity-discrimination and phenotype-discovery analysis rather than a real-time admission-only prediction model. We further performed an exploratory single-center temporal validation using a small post-2019 cohort. The purpose of this study was not to directly prove molecular inflammasome activation, but to explore whether routinely available clinical variables could identify a clinically inferred inflammatory injury phenotype biologically compatible with inflammasome-related inflammation associated with severe pediatric adenovirus pneumonia and provide a basis for future prospective biomarker validation (Collins et al., 2024). The overall study workflow is shown in **Figure 1**.

## 2 Materials and methods

### 2.1 Study design and participants

This was a retrospective observational study of hospitalized children diagnosed with adenovirus pneumonia at Hubei Maternal and Child Health Hospital between December 2018 and August 2019. This original cohort was used as the model-development cohort. The dataset included 82 patients, comprising 32 mild cases and 50 severe cases. Adenovirus infection was confirmed by direct immunofluorescence detection of adenovirus antigen in nasopharyngeal swab specimens.

Disease severity was classified according to pediatric community-acquired pneumonia criteria, integrating clinical symptoms, physical signs, imaging findings, respiratory compromise, and extrapulmonary complications (Subspecialty Group of Respiratory, the Society of Pediatrics, Chinese Medical Association et al., 2024). Severe pneumonia was defined by the presence of severe respiratory manifestations or systemic complications, including respiratory distress, hypoxemia or cyanosis, altered mental status, dehydration, extrapulmonary organ injury, or other clinically significant complications requiring intensive monitoring or escalation of care.

To explore the temporal generalizability of the frozen model, we additionally collected a small post-2019 single-center temporal validation cohort from the same institution. This exploratory cohort included 39 hospitalized children with adenovirus pneumonia, comprising 23 mild and 16 severe cases. The temporal validation cohort was not used for feature selection, model training, threshold selection, or model tuning. The frozen models developed from the original cohort were directly applied to this cohort without retraining. Because of the small sample size and single-center design, this analysis was considered exploratory and was not interpreted as definitive external validation.

The study was approved by the Institutional Ethics Committee of Hubei Maternal and Child Health Hospital (Approval No. 2026-056-01). Because this was a retrospective analysis of de-identified clinical data, the requirement for individual informed consent was waived by the ethics committee.

### 2.2 Data collection and preprocessing

Demographic, clinical, and laboratory variables were extracted from electronic medical records. Demographic variables included age and sex. Clinical variables included fever duration during the total febrile course, maximal body temperature before admission or within the first 24 hours after admission, wet rales documented on admission physical examination, and other admission-related clinical signs. Laboratory variables included white blood cell count, neutrophil count, lymphocyte count, hemoglobin, platelet count, CRP, alanine aminotransferase (ALT), AST, albumin, LDH, creatine kinase-MB (CK-MB), and the derived neutrophil-to-lymphocyte ratio (NLR).

Laboratory variables were obtained from the first available blood tests within 24 hours after admission whenever possible. Fever duration was defined as the total number of febrile days during the disease course and was therefore interpreted as a disease-burden variable rather than a strictly admission-available predictor. Accordingly, the machine-learning analysis was used for clinical severity discrimination and phenotype discovery rather than for real-time admission-only prediction. Maximal temperature was defined as the highest recorded body temperature before admission or within the first 24 hours after admission. Wet rales referred to rales documented during the admission physical examination. Variables reflecting post-admission disease progression, treatment response, or length of hospital stay were not included in the predictive models. Because fever duration reflected the total febrile course, the model should not be interpreted as a purely admission-based prediction tool.

Age strings were converted into months. Numeric variables recorded as text, such as values below the lower detection limit, were converted into numeric values using predefined conservative rules. Candidate variables were selected before modeling based on routine clinical availability, clinical relevance to pediatric adenovirus pneumonia severity, and biological plausibility related to systemic inflammation or tissue injury. No data-driven feature selection was performed using the outcome labels.

Missing values were imputed using median imputation within the modeling pipeline. Continuous variables were standardized using z-score normalization for algorithms sensitive to feature scaling. To avoid information leakage, all preprocessing steps, including imputation, IAI construction, and scaling, were performed within each cross-validation fold rather than before data splitting (Collins et al., 2024). For the exploratory temporal validation cohort, the same 14 raw input variables were extracted where available. The model-development pipeline was locked before validation. Therefore, the post-2019 cohort was only used for testing the frozen models, without retraining, feature reselection, threshold adjustment, or recalculation of preprocessing parameters. Missing-value imputation, z-score standardization, and IAI calculation were performed using parameters derived from the original development cohort.

Variables with substantial missingness were retained in the primary exploratory analysis if they were clinically meaningful. A sensitivity analysis excluding albumin and CK-MB was performed to evaluate whether model performance depended on variables with higher missingness.

### 2.3 Construction of the surrogate inflammasome-associated inflammatory injury index

Because direct inflammasome biomarkers were not available in this retrospective clinical dataset, we constructed a surrogate inflammasome-associated inflammatory injury index, termed the Inflammasome-associated Inflammatory Injury Index (IAI). The IAI was designed to approximate the clinical burden of systemic inflammation, fever response, and tissue injury that may occur downstream of inflammasome-related inflammatory pathways. It was developed as an exploratory research-derived composite index for phenotype characterization and model interpretation, not as a validated bedside scoring system. It should not be interpreted as a direct molecular measurement of NLRP3 inflammasome activation.

The IAI was calculated as the mean of five z-standardized variables: LDH, CRP, AST, NLR, and maximal temperature. Specifically, the IAI was defined as:


$$\text{IAI}=\frac{z\left(LDH\right)+z\left(CRP\right)+z\left(AST\right)+z\left(NLR\right)+z\left(maximal\;temperature\right)}{5}$$

where each z-score was calculated as the observed value minus the corresponding mean and divided by the corresponding standard deviation. For descriptive analyses and phenotype discovery in the model-development cohort, z-standardization was based on the development cohort. For cross-validation analyses, z-standardization and IAI construction were performed within each training fold to reduce information leakage. For the exploratory temporal validation cohort, IAI was calculated using the locked parameters derived from the original development cohort.

The biological rationale for variable selection was as follows. LDH reflects cellular injury and lytic damage; CRP reflects systemic acute-phase inflammation; AST reflects tissue injury and extrapulmonary involvement; NLR reflects innate immune predominance and relative lymphocyte suppression; and maximal temperature reflects pyrogenic inflammatory signaling (Schroder and Tschopp, 2010; Swanson et al., 2019). These variables were selected because they are routinely available in pediatric clinical practice and may capture clinically measurable consequences of inflammatory tissue injury. Higher IAI values indicate greater clinically inferred systemic inflammatory and tissue-injury burden that is biologically compatible with inflammasome-associated inflammation.

Given the exploratory nature of this index, additional sensitivity analyses are recommended in subsequent validation studies to compare models using original variables alone, IAI alone, and IAI combined with non-overlapping clinical predictors. Future prospective studies should further evaluate whether IAI correlates with direct inflammasome-related biomarkers, including NLRP3, caspase-1, interleukin-1β, interleukin-18, and gasdermin D.

### 2.4 Machine-learning models, internal validation, and exploratory temporal validation

Three supervised classifiers were evaluated for discriminating severe adenovirus pneumonia: logistic regression, support vector machine with radial basis function kernel (SVM-RBF), and random forest. Logistic regression was included as an interpretable linear benchmark. SVM-RBF was used to capture nonlinear decision boundaries. Random forest was selected as a tree-based ensemble method suitable for modeling nonlinear interactions in tabular clinical datasets (Cortes and Vapnik, 1995; Breiman, 2001; Pedregosa et al., 2011).

Model performance was assessed using repeated stratified 5-fold cross-validation with five repeats. Stratification was used to preserve the mild/severe class distribution in each fold. The primary evaluation metric was the area under the receiver-operating-characteristic curve (AUC). Additional metrics included area under the precision-recall curve (PR-AUC), accuracy, precision, recall, F1-score, and Brier score. The Brier score was used as a calibration-related measure of probabilistic prediction error, with lower values indicating better agreement between predicted probabilities and observed outcomes. Stratified 5-fold cross-validation was further performed to generate out-of-fold predictions for ROC curve construction and to evaluate model discrimination and probabilistic prediction error, including the Brier score.

To reduce the risk of optimistic bias, all preprocessing procedures, including missing-value imputation, IAI construction, and feature scaling, were embedded within the cross-validation pipeline. In each cross-validation iteration, imputation values, standardization parameters, and IAI-related z-score parameters were estimated only from the training fold and then applied to the corresponding validation fold.

To further explore model generalizability over time, we performed an exploratory single-center temporal validation using the post-2019 cohort described above. The model-development pipeline was locked before validation. Therefore, the post-2019 cohort was used only for testing the frozen models, without retraining, feature reselection, threshold adjustment, or recalculation of preprocessing parameters. Missing-value imputation, z-score standardization, and IAI calculation were performed using parameters derived from the original development cohort. Model discrimination, classification performance, and probabilistic prediction error were assessed using AUC, PR-AUC, accuracy, sensitivity, specificity, F1-score, and Brier score. Because this temporal validation cohort was small and derived from the same institution, it was interpreted as exploratory and not as definitive external validation.

### 2.5 Model interpretation using SHAP

The best-performing model was interpreted using SHAP values. SHAP provides additive feature-attribution values for individual predictions and allows both global feature ranking and patient-level interpretation (Lundberg and Lee, 2017; Lundberg et al., 2020). Mean absolute SHAP values were used to rank the overall importance of predictors. SHAP value distribution plots were used to visualize whether higher or lower feature values contributed to increased predicted probability of severe disease.

SHAP analysis was performed on the final random forest model refitted using the complete model-development cohort after internal performance evaluation. This analysis was intended to explain the fitted model structure rather than to provide an independent estimate of model performance. Because IAI was derived from several routine inflammatory and tissue-injury variables, its interpretation was considered complementary to, rather than independent from, its component variables. Therefore, SHAP results were interpreted as evidence of the predictive importance of an integrated inflammatory injury pattern, rather than as direct proof of molecular inflammasome activation.

Importantly, SHAP values indicate how much each feature contributed to model prediction within this dataset. They should not be interpreted as evidence that a variable causally drives severe disease, directly activates inflammasome pathways, or mediates disease progression. Accordingly, SHAP findings were described as model-based associations and hypothesis-generating signals.

### 2.6 Phenotype discovery based on IAI-related inflammatory injury variables

To identify clinically interpretable inflammatory injury phenotypes, unsupervised k-means clustering was performed using the z-standardized IAI-related variables: LDH, CRP, AST, NLR, and maximal temperature. These variables were selected before clustering because they represented the components of the surrogate IAI.

Given the modest sample size and the aim of identifying clinically interpretable subgroups, a two-cluster solution was used for the primary analysis. The cluster with the higher mean IAI was labeled the inflammatory injury-high phenotype, whereas the other cluster was labeled the inflammatory injury-low phenotype. Severe-case enrichment between phenotypes was compared using Fisher’s exact test.

The clustering variables were routine clinical and laboratory indicators, not direct inflammasome biomarkers. Accordingly, the identified phenotype was interpreted as a clinically inferred inflammatory injury phenotype rather than molecular evidence of NLRP3 inflammasome activation, caspase-1 activation, interleukin-1β/interleukin-18 signaling, gasdermin D-mediated pyroptosis, or any other specific inflammasome pathway.

### 2.7 Statistical analysis

Continuous variables were summarized as mean ± standard deviation and median with interquartile range. Between-group comparisons were performed using the Mann–Whitney U test because several variables showed skewed distributions or unequal variance. Categorical variables were summarized as counts and percentages and compared using the chi-square test or Fisher’s exact test, as appropriate.

Spearman correlation analysis was performed to evaluate monotonic associations among disease severity, fever burden, laboratory variables, and IAI. Disease severity was coded as a binary variable, with mild disease coded as 0 and severe disease coded as 1. A two-sided p value< 0.05 was considered statistically significant. Because this was an exploratory retrospective study, p values for baseline comparisons were not adjusted for multiple testing and should be interpreted descriptively.

For the exploratory temporal validation cohort, model discrimination was assessed using the area under the receiver-operating-characteristic curve (AUC) and the area under the precision-recall curve (PR-AUC). Classification performance was evaluated using accuracy, precision, sensitivity, specificity, and F1-score at the fixed probability threshold of 0.5. The Brier score was used to describe probabilistic prediction error. Bootstrap resampling was used to estimate the 95% confidence interval of the AUC. A calibration plot was generated for descriptive visualization only, given the small sample size of the temporal validation cohort.

All analyses were conducted using Python with scikit-learn and SHAP. The study was reported with reference to TRIPOD+AI recommendations for clinical prediction models using regression or machine-learning methods. No model retraining, threshold adjustment, or variable reselection was performed in the exploratory temporal validation cohort.

## 3 Results

### 3.1 Baseline characteristics and clinical differences between mild and severe cases

The study cohort included 82 children with adenovirus pneumonia, comprising 32 mild cases and 50 severe cases. Baseline clinical and laboratory characteristics are summarized in **Table 1**. Severe cases were significantly younger than mild cases (26.84 ± 20.80 vs. 43.69 ± 30.39 months, p = 0.004). Male sex was more frequent in the severe group, although the difference did not reach statistical significance (70.0% vs. 50.0%, p = 0.071).

**Table 1:** Baseline clinical and laboratory characteristics of children with mild and severe adenovirus pneumonia.

Variable	Mild (n=32)	Severe (n=50)	P value
Age, months	43.69 ± 30.39; 38.00 (21.00–58.00)	26.84 ± 20.80; 20.00 (12.00–37.00)	0.004
Male sex	16/32 (50.0%)	35/50 (70.0%)	0.071
Fever duration, days	5.68 ± 2.06; 5.00 (5.00–7.00)	11.65 ± 5.91; 10.00 (9.00–15.50)	<0.001
Maximal temperature, °C	39.59 ± 0.54; 39.60 (39.23–39.98)	40.11 ± 0.61; 40.20 (39.80–40.50)	<0.001
Hemoglobin, g/L	122.25 ± 9.75; 123.50 (114.75–128.00)	114.72 ± 10.56; 115.50 (107.00–122.00)	0.005
Platelets, ×10^9^/L	228.75 ± 67.26; 226.50 (186.50–272.00)	244.86 ± 119.36; 222.50 (170.75–290.00)	0.955
CRP, mg/L	22.09 ± 26.14; 12.19 (7.00–28.66)	34.80 ± 44.77; 13.35 (5.77–46.74)	0.573
ALT, U/L	14.11 ± 5.81; 12.20 (10.68–16.05)	27.95 ± 30.02; 15.15 (12.07–30.68)	0.018
AST, U/L	35.41 ± 9.56; 35.30 (28.30–40.30)	61.00 ± 41.95; 45.60 (35.20–71.00)	<0.001
LDH, U/L	316.54 ± 104.67; 295.00 (250.50–343.25)	542.14 ± 320.90; 394.00 (337.00–712.50)	<0.001
CK-MB, U/L	30.02 ± 11.53; 26.00 (23.30–35.20)	45.24 ± 20.01; 41.40 (29.20–62.20)	0.002
Neutrophil-to-lymphocyte ratio	3.79 ± 5.60; 2.18 (1.06–5.03)	2.86 ± 2.01; 2.40 (1.62–3.67)	0.973
IAI	-0.29 ± 0.46; -0.36 (-0.53 to -0.12)	0.18 ± 0.56; 0.02 (-0.18 to 0.45)	<0.001
Wet rales, n (%)	5/32 (15.6%)	28/50 (56.0%)	<0.001

Values are presented as mean ± standard deviation and median (interquartile range), except categorical variables, which are shown as n/N (%). Continuous variables were compared using the Mann–Whitney U test. Categorical variables were compared using the chi-square test or Fisher’s exact test, as appropriate. Fever duration refers to the total number of febrile days during the disease course. Maximal temperature refers to the highest recorded body temperature before admission or within the first 24 hours after admission. Wet rales refer to rales documented during the admission physical examination. IAI, Inflammasome-associated Inflammatory Injury Index; NLR, neutrophil-to-lymphocyte ratio; CRP, C-reactive protein; ALT, alanine aminotransferase; AST, aspartate aminotransferase; LDH, lactate dehydrogenase; CK-MB, creatine kinase-MB.

Children with severe adenovirus pneumonia showed a more prolonged and intense febrile course. Total fever duration during the disease course was significantly longer in severe cases than in mild cases (11.65 ± 5.91 vs. 5.68 ± 2.06 days, p< 0.001). Maximal temperature before admission or within the first 24 hours after admission was also higher in the severe group (40.11 ± 0.61 vs. 39.59 ± 0.54 °C, p< 0.001). Admission wet rales were more frequently observed in severe cases than in mild cases (56.0% vs. 15.6%, p< 0.001), indicating more prominent lower respiratory tract involvement at presentation.

Laboratory findings suggested a systemic inflammatory and tissue-injury pattern in severe disease. Hemoglobin levels were lower in severe cases (114.72 ± 10.56 vs. 122.25 ± 9.75 g/L, p = 0.005). ALT, AST, LDH, and CK-MB levels were higher in the severe group, with particularly significant differences in AST, LDH, and CK-MB. In contrast, platelet count, CRP, and NLR did not differ significantly between the two groups when evaluated as individual variables.

The surrogate Inflammasome-associated Inflammatory Injury Index (IAI) was significantly higher in severe cases than in mild cases (0.18 ± 0.56 vs. −0.29 ± 0.46, p< 0.001). This finding suggests that the integrated pattern of fever burden, systemic inflammation, innate immune imbalance, and tissue injury was more pronounced in children with severe adenovirus pneumonia, even though the IAI should be interpreted as a surrogate clinical index rather than a direct molecular measurement of inflammasome activation.

### 3.2 Correlation structure was consistent with an integrated inflammatory injury pattern

**Figure 2:**
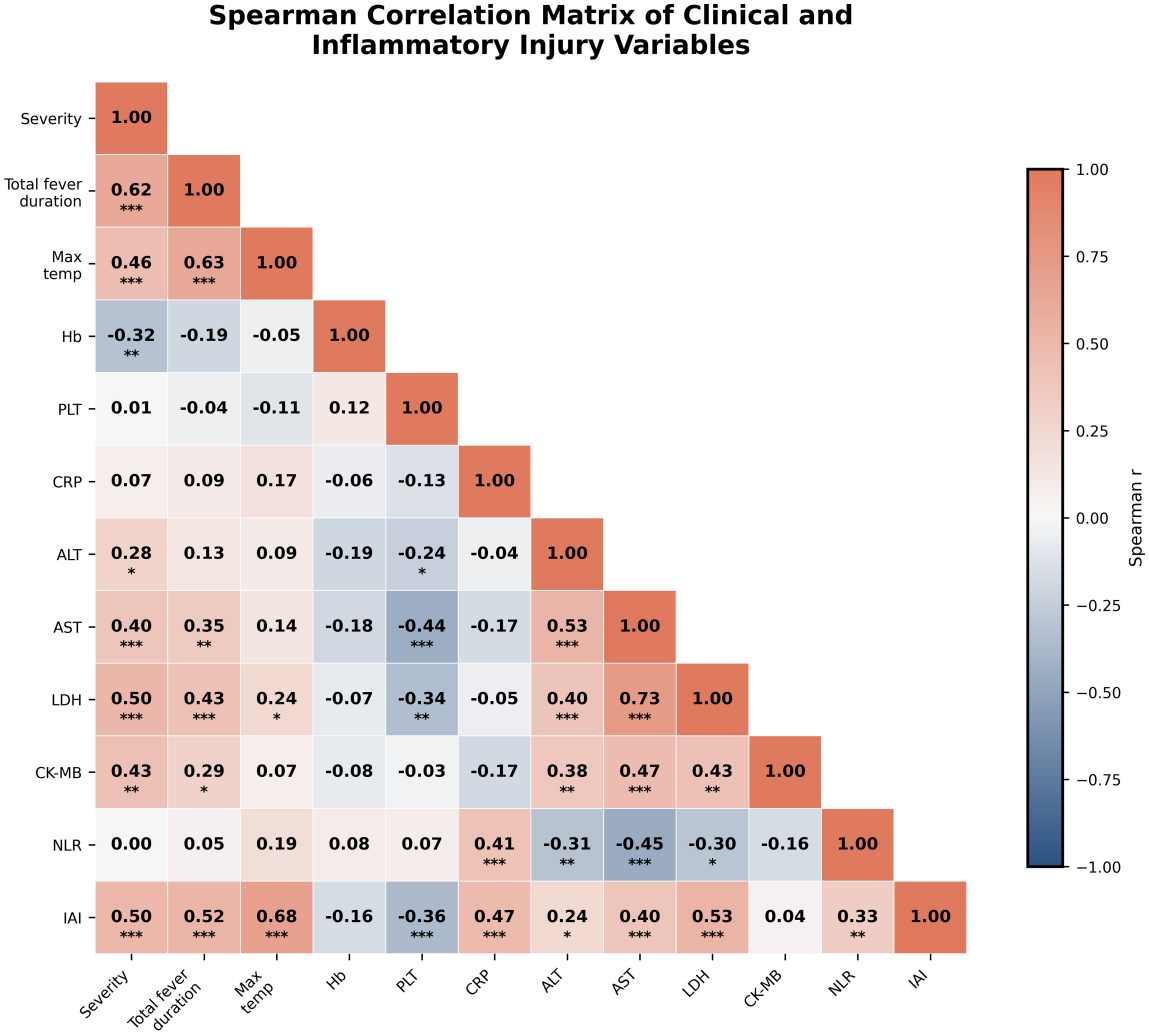
Spearman correlation matrix of clinical and inflammatory injury variables. The matrix shows Spearman correlation coefficients among disease severity, fever-related variables, hematological indices, inflammatory markers, tissue-injury markers, and IAI. Severity was coded as 0 = mild and 1 = severe. Positive correlations are shown in red, and negative correlations are shown in blue. Asterisks indicate statistical significance. IAI, Inflammasome-associated Inflammatory Injury Index; Hb, hemoglobin; PLT, platelet count; CRP, C-reactive protein; ALT, alanine aminotransferase; AST, aspartate aminotransferase; LDH, lactate dehydrogenase; CK-MB, creatine kinase-MB; NLR, neutrophil-to-lymphocyte ratio; Max temp, maximal temperature. *p< 0.05, **p< 0.01, ***p< 0.001.

Spearman correlation analysis was performed to explore the relationships among disease severity, fever-related variables, tissue-injury markers, and the surrogate IAI (**Figure 2**). Disease severity was coded as a binary variable, with mild disease coded as 0 and severe disease coded as 1.

Severity showed positive correlations with total fever duration, maximal temperature, and IAI, supporting the clinical relevance of fever burden and integrated inflammatory injury in severe adenovirus pneumonia. The IAI correlated with its component variables, including LDH, CRP, AST, NLR, and maximal temperature, indicating that it functioned as a compact composite summary of systemic inflammation, pyrogenic response, and tissue injury. LDH and AST also showed a positive correlation, consistent with their shared reflection of cellular injury and extrapulmonary involvement.

Together, these correlation patterns were consistent with a clinically coherent inflammatory injury axis in pediatric adenovirus pneumonia. Importantly, these associations do not prove molecular inflammasome activation, but they provide a rationale for using the IAI as a surrogate clinical measure to explore clinically inferred inflammatory injury phenotypes that may be biologically compatible with inflammasome-associated inflammation.

### 3.3 Machine-learning models showed meaningful internal discrimination for severe adenovirus pneumonia

Three supervised models were developed for clinical discrimination of severe adenovirus pneumonia, including logistic regression, support vector machine with radial basis function kernel, and random forest. Model performance was evaluated using repeated stratified 5-fold cross-validation with five repeats, with all preprocessing steps embedded within the cross-validation pipeline.

**Figure 3:**
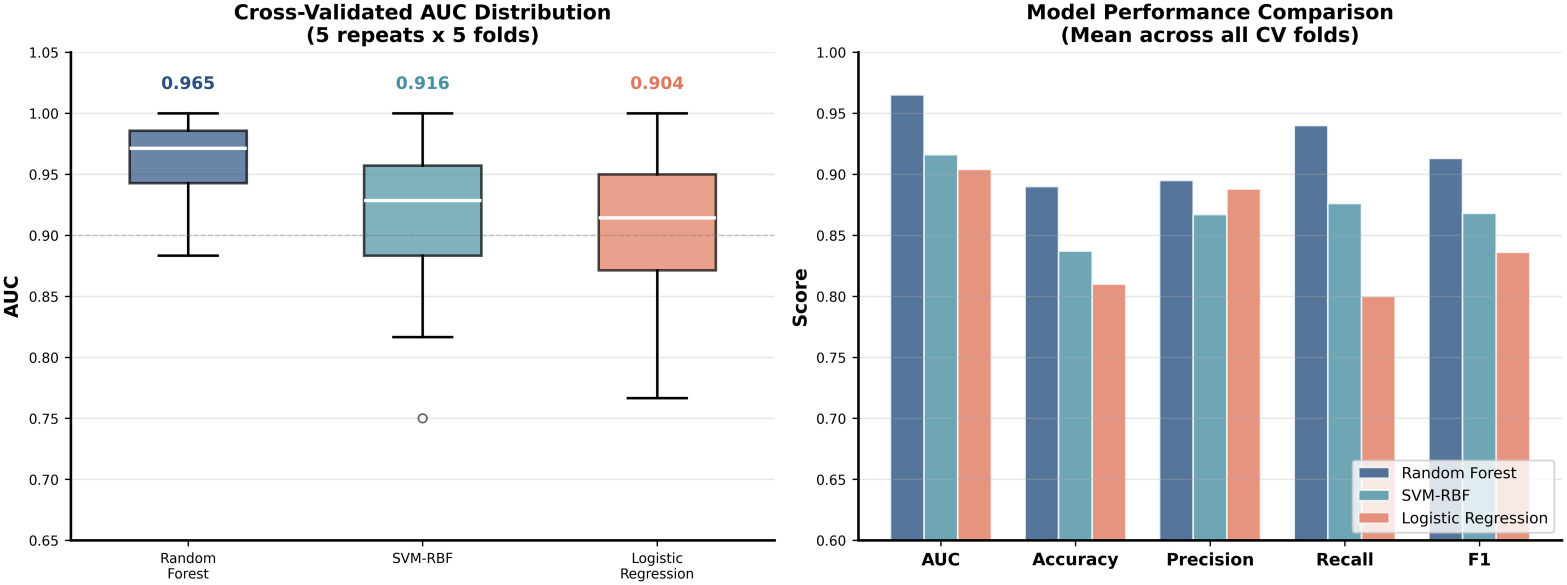
Summary of repeated cross-validated model performance. Left: AUC distribution across repeated stratified 5-fold cross-validation with five repeats. Values above the boxplots indicate the mean AUC of each model. Right: comparison of mean performance metrics across all cross-validation folds, including AUC, accuracy, precision, recall, and F1-score. All preprocessing procedures, including missing-value imputation, IAI construction, and feature scaling, were embedded within each training fold. Random forest showed the best overall internal performance among the evaluated models. AUC, area under the receiver-operating-characteristic curve; IAI, Inflammasome-associated Inflammatory Injury Index; SVM-RBF, support vector machine with radial basis function kernel; CV, cross-validation.

All three models showed meaningful internal discrimination for severe adenovirus pneumonia (**Table 2**). Among them, random forest achieved the best overall performance, with a repeated cross-validated AUC of 0.965 ± 0.031, PR-AUC of 0.980 ± 0.018, accuracy of 0.890, precision of 0.895, recall of 0.940, and F1-score of 0.913. SVM-RBF and logistic regression also showed good discrimination, with AUC values of 0.916 ± 0.062 and 0.904 ± 0.064, respectively. The distribution of cross-validated AUC values and the comparison of mean performance metrics across models are shown in **Figure 3**.

**Table 2:** Repeated stratified 5-fold cross-validation performance of the supervised machine-learning models.

Model	AUC, mean ± SD	PR-AUC, mean ± SD	Accuracy	Precision	Recall	F1-score
Random forest	0.965 ± 0.031	0.980 ± 0.018	0.890	0.895	0.940	0.913
SVM-RBF	0.916 ± 0.062	0.949 ± 0.040	0.837	0.867	0.876	0.868
Logistic regression	0.904 ± 0.064	0.944 ± 0.037	0.810	0.888	0.800	0.836

Model performance was evaluated using repeated stratified 5-fold cross-validation with five repeats. Values are presented as mean ± standard deviation for AUC and PR-AUC, and as mean values across all cross-validation folds for accuracy, precision, recall, and F1-score. All preprocessing procedures, including missing-value imputation and feature scaling, were performed within each training fold to reduce information leakage. AUC, area under the receiver-operating-characteristic curve; PR-AUC, area under the precision-recall curve; SVM-RBF, support vector machine with radial basis function kernel.

**Figure 4:**
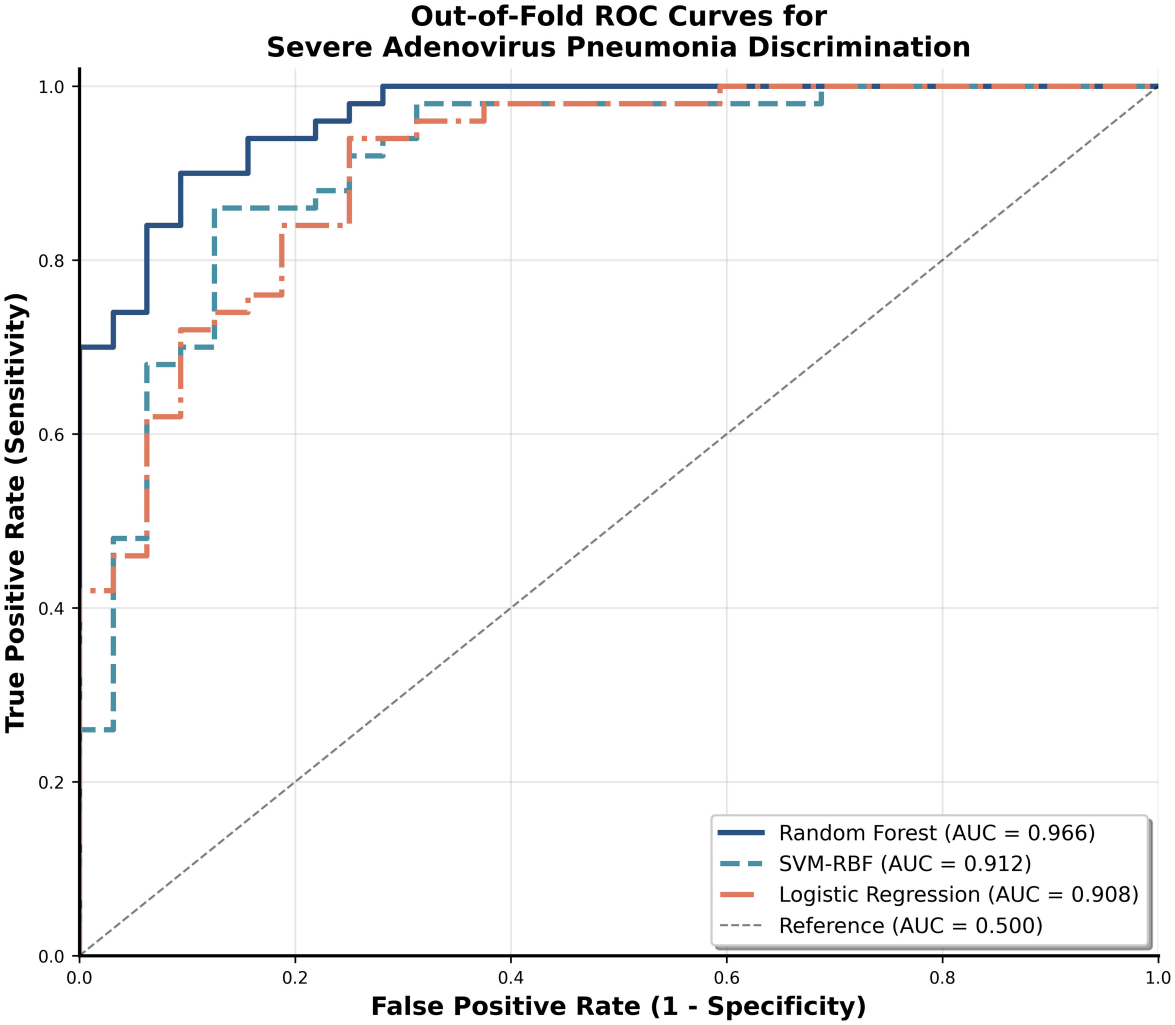
Out-of-fold ROC curves for severe adenovirus pneumonia discrimination. ROC curves were generated from five-fold out-of-fold predictions within the same cohort. Random forest achieved the highest out-of-fold AUC, followed by SVM-RBF and logistic regression. These results represent internal out-of-fold evaluation and should not be interpreted as external validation. ROC, receiver-operating-characteristic; AUC, area under the receiver-operating-characteristic curve; SVM-RBF, support vector machine with radial basis function kernel.

To further visualize model discrimination, a stratified 5-fold out-of-fold prediction analysis was performed. In this analysis, random forest again showed the highest internal performance, apparent with an out-of-fold AUC of 0.966, PR-AUC of 0.978, accuracy of 0.890, F1-score of 0.913, and Brier score of 0.088 (**Table 3**; **Figure 4**). Logistic regression and SVM-RBF achieved out-of-fold AUC values of 0.908 and 0.912, respectively.

**Table 3:** Five-fold out-of-fold performance for severe adenovirus pneumonia discrimination.

Model	OOF AUC	OOF PR-AUC	OOF accuracy	OOF F1-score	Brier score
Random forest	0.966	0.978	0.890	0.913	0.088
Logistic regression	0.908	0.937	0.805	0.833	0.132
SVM-RBF	0.912	0.935	0.841	0.869	0.115

Out-of-fold predictions were generated within the same cohort using stratified 5-fold cross-validation. The Brier score was used as a calibration-related measure of probabilistic prediction error, with lower values indicating better agreement between predicted probabilities and observed outcomes. These results represent internal out-of-fold evaluation and should not be interpreted as external validation. OOF, out-of-fold; AUC, area under the receiver-operating-characteristic curve; PR-AUC, area under the precision-recall curve; SVM-RBF, support vector machine with radial basis function kernel.

These findings suggest that routine clinical and laboratory variables contained substantial information for distinguishing severe from mild adenovirus pneumonia in this cohort. Given the modest sample size and the exploratory nature of the single-center temporal validation cohort, these performance estimates should be interpreted cautiously and should not be considered evidence of clinical deployment readiness.

### 3.4 SHAP interpretation identified total fever duration and IAI as leading contributors to model prediction

**Figure 5:**
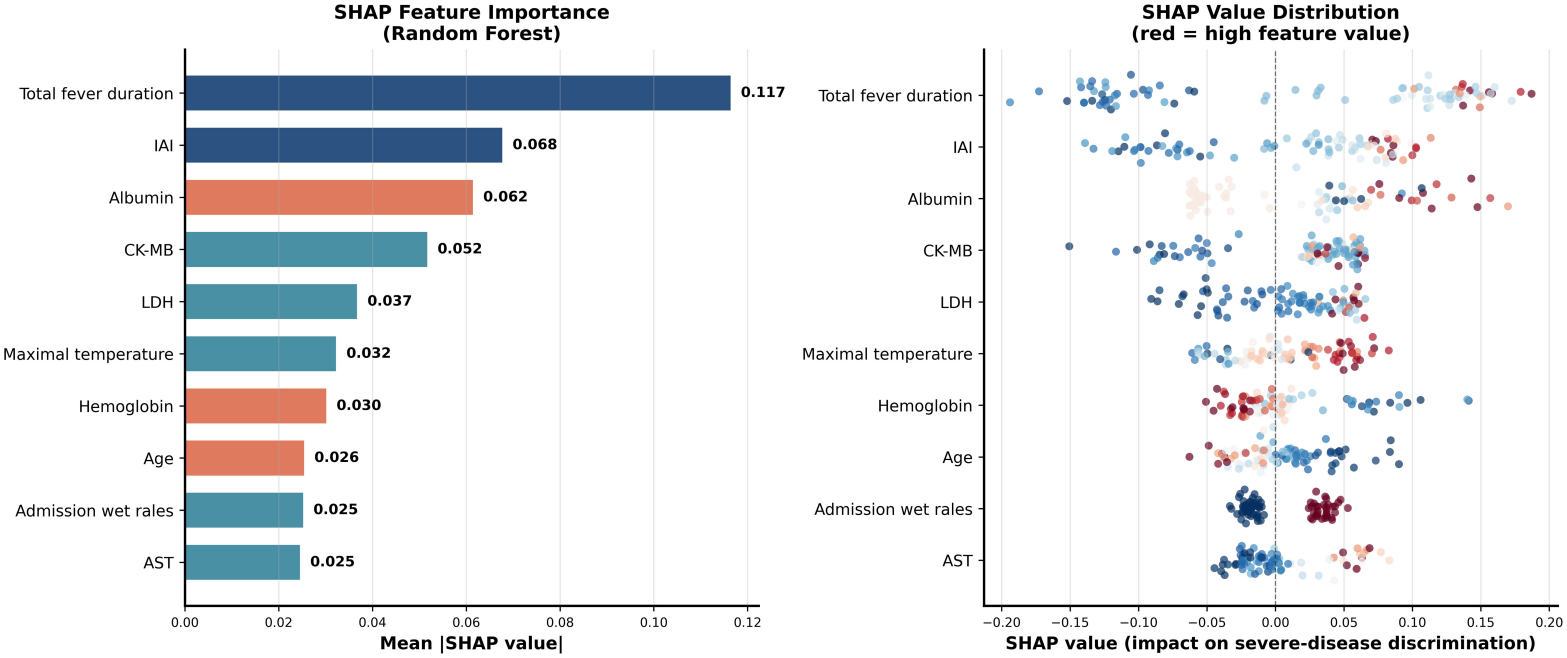
SHAP-based interpretation of the random forest model. Left: mean absolute SHAP values showing global feature importance in the random forest model. Right: SHAP value distribution colored by feature value, with red indicating higher feature values and blue indicating lower feature values. Higher SHAP values indicate greater contribution to the predicted probability of severe adenovirus pneumonia within this model. Total fever duration and IAI were the two leading contributors to model prediction. SHAP values should not be interpreted as evidence of causality or direct molecular inflammasome activation. IAI, Inflammasome-associated Inflammatory Injury Index; SHAP, Shapley additive explanations; CK-MB, creatine kinase-MB; LDH, lactate dehydrogenase; AST, aspartate aminotransferase.

To improve model transparency, SHAP analysis was applied to the random forest model. Mean absolute SHAP values were used to rank the global contribution of each variable to severe disease discrimination (**Figure 5**).

Total fever duration was the most influential contributor, followed by the surrogate Inflammasome-associated Inflammatory Injury Index. This indicates that both prolonged total febrile course and an integrated inflammatory injury signal contributed prominently to model-based severity discrimination. Other variables contributing to prediction included albumin, CK-MB, AST, age, LDH, and admission wet rales.

The SHAP distribution plot further indicated that higher total fever duration and higher IAI generally shifted predictions toward severe disease. This pattern is clinically plausible because persistent fever may reflect sustained inflammatory signaling, while IAI integrates fever burden, systemic inflammation, innate immune imbalance, and tissue injury. Similarly, higher AST, LDH, and CK-MB values may reflect extrapulmonary tissue injury or systemic involvement in severe adenovirus pneumonia.

Importantly, the SHAP ranking should not be interpreted as direct evidence that molecular inflammasome activation was measured in this cohort. Rather, the prominence of IAI suggests that an integrated clinically inferred inflammatory injury pattern, captured by routine clinical variables, provided important predictive information beyond individual respiratory signs alone. SHAP values indicate feature contributions to model prediction within this dataset and do not establish that these variables causally drive severe disease, inflammasome activation, or disease progression.

### 3.5 Unsupervised clustering identified a clinically inferred inflammatory injury-high phenotype enriched for severe disease

To explore whether routine IAI-related variables could identify clinically meaningful inflammatory injury phenotypes, unsupervised k-means clustering was performed using z-standardized LDH, CRP, AST, NLR, and maximal temperature. These variables were selected because they represented the components of the surrogate IAI and captured fever response, systemic inflammation, innate immune imbalance, and tissue injury.

**Figure 6:**
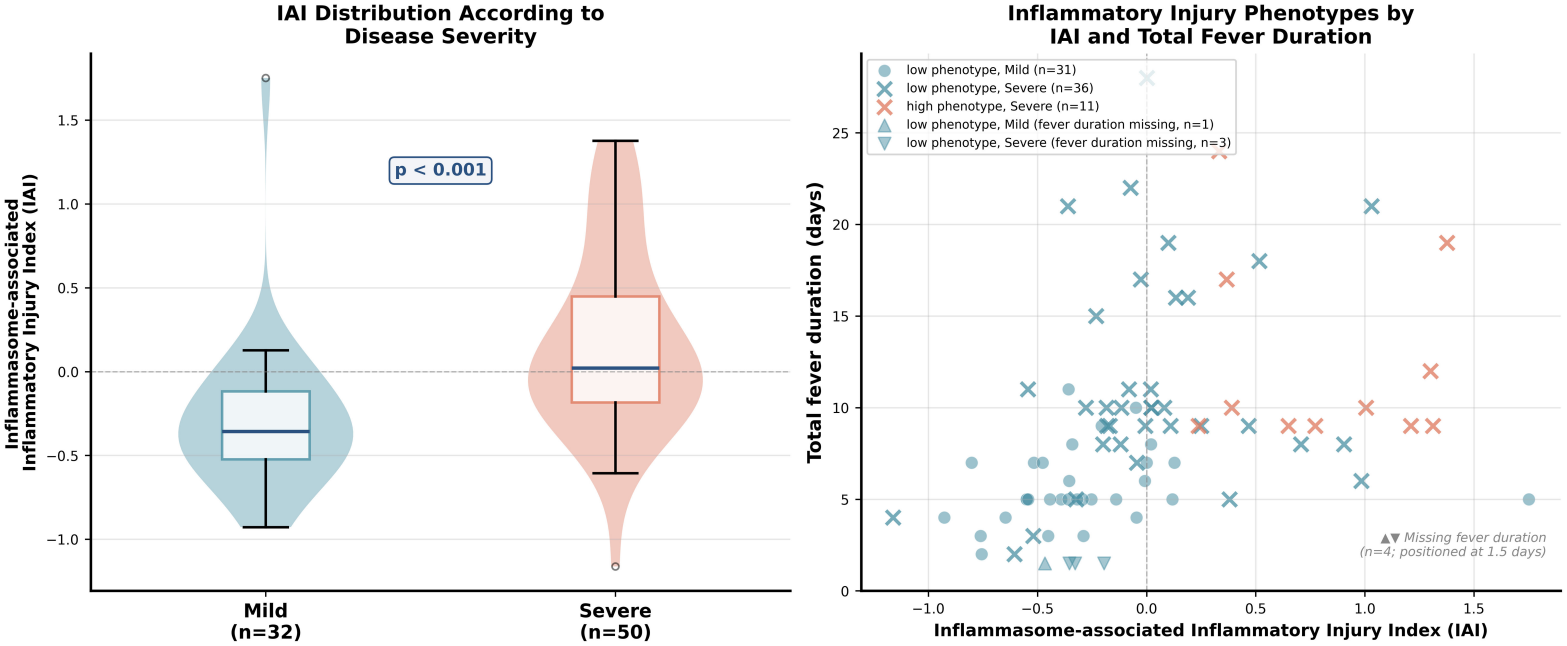
IAI distribution and clinically inferred inflammatory injury phenotype discovery. Left: distribution of the Inflammasome-associated Inflammatory Injury Index according to disease severity. Severe cases showed significantly higher IAI values than mild cases. Right: scatter plot of IAI and total fever duration according to inflammatory injury phenotype and clinical severity. Colors indicate inflammatory injury phenotypes, and point shapes indicate clinical severity. The inflammatory injury-high phenotype included 11 severe cases. In the inflammatory injury-low phenotype, 32 mild and 39 severe cases were identified; fever duration was missing in 1 mild case and 7 severe cases. For visualization only, patients with missing fever duration were positioned at 1.5 days. The identified phenotype should be interpreted as a clinically inferred inflammatory injury phenotype rather than direct molecular evidence of inflammasome activation. IAI, Inflammasome-associated Inflammatory Injury Index.

A two-cluster solution separated patients into an inflammatory injury-high phenotype and an inflammatory injury-low phenotype (**Table 4**; **Figure 6**). The inflammatory injury-high phenotype included 11 patients and was characterized by markedly higher mean IAI, longer total fever duration, higher LDH, higher AST, and higher CRP values. All 11 patients in this phenotype had severe adenovirus pneumonia. In contrast, the inflammatory injury-low phenotype included 71 patients, of whom 39 had severe disease and 32 had mild disease. Severe-case enrichment differed significantly between the two phenotypes by Fisher’s exact test (100.0% vs. 54.9%, p = 0.005).

**Table 4:** Clinically inferred inflammatory injury phenotypes identified by unsupervised clustering.

Phenotype	n	Severe cases	Mean IAI	Fever duration, days	LDH, U/L	AST, U/L	CRP, mg/L
Inflammatory injury-high	11	11 (100.0%)	0.82	12.45	1023.21	110.11	39.96
Inflammatory injury-low	71	39 (54.9%)	-0.13	8.66	348.66	40.40	27.48

Phenotypes were derived by k-means clustering of z-standardized LDH, CRP, AST, NLR, and maximal temperature. The cluster with the higher mean IAI was labeled the inflammatory injury-high phenotype, whereas the other cluster was labeled the inflammatory injury-low phenotype. Severe-case enrichment between phenotypes was compared using Fisher’s exact test. The identified phenotypes should be interpreted as clinically inferred inflammatory injury patterns rather than direct molecular evidence of inflammasome activation. IAI, Inflammasome-associated Inflammatory Injury Index; LDH, lactate dehydrogenase; CRP, C-reactive protein; AST, aspartate aminotransferase; NLR, neutrophil-to-lymphocyte ratio.

This clustering analysis suggests that a subset of children with adenovirus pneumonia exhibited a distinct systemic inflammatory injury pattern. Importantly, the phenotype was derived without using the severity label, indicating that this pattern was identifiable from routine fever, inflammation, and tissue-injury variables alone. It should be emphasized that the clustering variables were routine clinical and laboratory indicators, not direct inflammasome biomarkers. Accordingly, the inflammatory injury-high phenotype should be interpreted as a clinically inferred inflammatory injury phenotype rather than molecular evidence of NLRP3 inflammasome activation, caspase-1 activation, interleukin-1β/interleukin-18 signaling, gasdermin D-mediated pyroptosis, or any other specific inflammasome pathway.

### 3.6 Sensitivity analysis excluding variables with substantial missingness

Because albumin and CK-MB had substantial missingness but contributed to the primary model, a sensitivity analysis was performed after excluding both variables. The same repeated stratified 5-fold cross-validation strategy was applied.

After removal of albumin and CK-MB, random forest remained the best-performing model, with an AUC of 0.919 ± 0.048, PR-AUC of 0.952 ± 0.031, accuracy of 0.843, and F1-score of 0.874 (**Table 5**). SVM-RBF and logistic regression also maintained acceptable discrimination, with AUC values of 0.897 ± 0.069 and 0.895 ± 0.071, respectively.

**Table 5:** Sensitivity analysis excluding variables with substantial missingness.

Sensitivity model	AUC, mean ± SD	PR-AUC, mean ± SD	Accuracy	Precision	Recall	F1-score
Random forest (no ALB/CK-MB)	0.919 ± 0.048	0.952 ± 0.031	0.843	0.868	0.892	0.874
SVM-RBF (no ALB/CK-MB)	0.897 ± 0.069	0.939 ± 0.042	0.806	0.837	0.860	0.842
Logistic regression (no ALB/CK-MB)	0.895 ± 0.071	0.938 ± 0.041	0.796	0.875	0.788	0.824

This sensitivity analysis used the same repeated stratified 5-fold cross-validation strategy as the primary analysis but excluded albumin and CK-MB because of substantial missingness. Values are presented as mean ± standard deviation for AUC and PR-AUC, and as mean values for accuracy, precision, recall, and F1-score across all cross-validation folds. All preprocessing procedures, including missing-value imputation, IAI construction, and feature scaling, were embedded within each training fold. AUC, area under the receiver-operating-characteristic curve; PR-AUC, area under the precision-recall curve; CK-MB, creatine kinase-MB; SVM-RBF, support vector machine with radial basis function kernel.

Although model performance decreased compared with the primary exploratory model, discrimination remained meaningful. This finding suggests that the prediction framework was not solely dependent on variables with substantial missingness. However, the performance reduction also indicates that albumin and CK-MB may contain clinically relevant information, and future prospective studies should collect these variables more systematically to minimize missing data. This analysis was considered an internal sensitivity analysis and should not be interpreted as external validation.

### 3.7 Exploratory single-center temporal validation

To explore whether the frozen models retained discriminative ability in later cases from the same institution, an exploratory single-center temporal validation was performed using a post-2019 cohort. This cohort included 39 hospitalized children with adenovirus pneumonia, comprising 23 mild and 16 severe cases. The temporal validation cohort was used only for model testing. No model retraining, feature reselection, threshold adjustment, or recalculation of preprocessing parameters was performed.

In this exploratory temporal validation cohort, the frozen random forest model achieved the best overall performance, with an AUC of 0.840, PR-AUC of 0.831, accuracy of 0.744, sensitivity of 0.750, specificity of 0.739, F1-score of 0.706, and Brier score of 0.173 (**Supplementary Table 1**). SVM-RBF and logistic regression achieved AUC values of 0.796 and 0.772, respectively. The ROC curve and descriptive calibration plot are provided in **Supplementary Figures 1**, **2**.

These findings suggest that the random forest model retained moderate discriminative ability in a small temporally distinct cohort. However, because this cohort was small and derived from the same institution, the analysis should be interpreted as exploratory temporal validation rather than definitive external validation. Larger prospective multicenter cohorts are still required to evaluate model generalizability, calibration, and clinical utility.

## 4 Discussion

In this retrospective cohort of children with adenovirus pneumonia, we developed a retrospective interpretable machine-learning framework for clinical severity discrimination and inflammatory phenotype stratification and explored its relationship with a clinically inferred inflammasome-associated inflammatory injury phenotype. Three main findings emerged. First, routine clinical and laboratory variables provided meaningful internal discrimination for severe adenovirus pneumonia, with random forest achieving the best performance among the evaluated models. Second, SHAP interpretation identified total fever duration and the surrogate Inflammasome-associated Inflammatory Injury Index (IAI) as the two leading contributors to model prediction. Third, unsupervised clustering based on IAI-related variables identified an inflammatory injury-high phenotype that was strongly enriched for severe disease. In addition, exploratory single-center temporal validation in a small post-2019 cohort suggested that the frozen random forest model retained moderate discriminative ability, although this analysis should not be interpreted as definitive external validation. Together, these findings suggest that a subset of children with severe adenovirus pneumonia may exhibit a clinically detectable systemic inflammatory injury pattern that is biologically compatible with inflammasome-related pathophysiology, but not direct molecular evidence of inflammasome activation.

Total fever duration was the most influential contributor to the random forest model. This finding is clinically plausible because severe adenovirus pneumonia in children is often characterized by prolonged high fever and intense systemic inflammation. In the present cohort, severe cases had a markedly longer total fever duration during the disease course and higher maximal temperature before admission or within the first 24 hours after admission. Fever is a nonspecific but important clinical manifestation of host inflammatory activation. From a mechanistic perspective, inflammasome-related cytokines, particularly IL-1β and IL-18, are closely involved in pyrogenic and inflammatory signaling (Dinarello, 2009; Man et al., 2017). Therefore, the strong contribution of fever duration is consistent with the hypothesis that sustained inflammatory activation contributes to severe adenovirus pneumonia. However, fever can also be driven by multiple innate immune and cytokine pathways; thus, it should not be interpreted as a specific indicator of inflammasome activation. In addition, because fever duration in this study reflected the total febrile course rather than a variable strictly available at initial presentation, its predictive contribution should be interpreted in the context of retrospective severity discrimination and disease-burden characterization, not real-time admission-only prediction.

The IAI was developed as a pragmatic surrogate index to summarize routine clinical markers reflecting fever burden, systemic inflammation, innate immune imbalance, and tissue injury. It should be regarded as an exploratory research-derived composite index for phenotype characterization and model interpretation, rather than a validated bedside scoring system. Its components were selected because LDH reflects cellular injury and lytic damage, CRP reflects acute-phase systemic inflammation, AST reflects tissue injury and extrapulmonary involvement, NLR reflects innate immune predominance and relative lymphocyte suppression, and maximal temperature reflects pyrogenic inflammatory signaling. In this cohort, IAI was significantly higher in severe cases and ranked second in SHAP importance (Gabay and Kushner, 1999; Dinarello, 2009; Schroder and Tschopp, 2010; Swanson et al., 2019). This suggests that the combined inflammatory injury signal may carry more clinically relevant information than some individual biomarkers alone. Notably, CRP and NLR did not differ significantly between mild and severe cases when evaluated separately, whereas their integration with LDH, AST, and temperature into the IAI revealed a stronger disease-associated pattern. This supports the value of composite feature engineering when the underlying biological process is multidimensional. Importantly, the IAI remains an indirect clinical surrogate. It cannot distinguish between inflammasome-mediated inflammation and other inflammatory pathways, and it should not be interpreted as direct evidence of NLRP3 inflammasome activation, caspase-1 activation, interleukin-1β/interleukin-18 signaling, or gasdermin D-mediated pyroptosis.

The relationship between IAI and inflammasome biology should be interpreted carefully. In addition, recent mechanistic studies and reviews have highlighted that NLRP3 inflammasome activation is not a single-step process but is controlled by multilayered regulatory mechanisms, including transcriptional priming, post-translational modification, licensing, and inflammasome assembly. These complex regulatory networks further support the need for direct molecular validation when attempting to link clinical inflammatory patterns to inflammasome biology (Liu et al., 2024; Paik et al., 2025). Experimental studies have shown that adenovirus can activate inflammasome pathways, including NLRP3 inflammasome activation during viral membrane penetration and HAdV-7 protein-mediated inflammasome assembly. Adenovirus-associated pulmonary inflammatory injury has also been linked to inflammasome activation and macrophage pyroptosis. These findings provide biological plausibility for exploring clinically inferred inflammatory injury patterns in severe adenovirus pneumonia. In the present study, however, NLRP3, caspase-1, IL-1β, IL-18, gasdermin D, and pyroptotic cell death were not directly measured. Therefore, the IAI should be understood as a clinically accessible surrogate of systemic inflammatory and tissue-injury burden that may be compatible with inflammasome-associated inflammation, rather than a molecular readout of inflammasome activation. Accordingly, the term “inflammasome-associated” in this study refers to a biologically motivated clinical inference and should not be interpreted as direct confirmation of inflammasome pathway activation in individual patients.

The unsupervised phenotype analysis further supports the potential clinical relevance of this inflammatory injury pattern. Clustering based only on LDH, CRP, AST, NLR, and maximal temperature identified an inflammatory injury-high phenotype comprising 11 patients, all of whom had severe adenovirus pneumonia. Importantly, disease severity labels were not used to generate the clusters, suggesting that this phenotype was discoverable from routine inflammatory and tissue-injury variables alone. This finding raises the possibility that severe adenovirus pneumonia is clinically heterogeneous: some patients may present with a particularly intense systemic inflammatory injury state, whereas others may develop severe disease through different combinations of respiratory compromise, host vulnerability, viral burden, or extrapulmonary complications. Future studies incorporating direct inflammasome biomarkers are needed to determine whether this inflammatory injury-high phenotype corresponds to true molecular inflammasome activation.

However, the clinical outcome implications of this phenotype remain uncertain. In the present retrospective dataset, outcomes such as pediatric intensive care unit admission, mechanical ventilation, duration of oxygen therapy, complications, mortality, and long-term pulmonary sequelae were not systematically available. Therefore, the inflammatory injury-high phenotype should be interpreted as a severity-enriched inflammatory injury subgroup rather than a validated prognostic subgroup. Future prospective studies should evaluate whether this phenotype is associated with clinically meaningful outcomes and whether it identifies patients who may benefit from closer monitoring or mechanism-guided biomarker assessment.

From a methodological perspective, this study highlights the potential value of combining clinically motivated feature engineering with interpretable machine learning (Breiman, 2001; Lundberg and Lee, 2017; Lundberg et al., 2020; Collins et al., 2024). Random forest achieved the best internal performance, which may reflect its ability to capture nonlinear relationships among age, fever duration, respiratory signs, inflammatory markers, and tissue-injury variables. To reduce optimistic bias, missing-value imputation, IAI construction, and feature scaling were embedded within each cross-validation fold rather than being performed before data splitting. However, prediction performance alone is insufficient for clinical translation, particularly in small retrospective datasets. Therefore, model discrimination was complemented by out-of-fold prediction and the Brier score as a calibration-related measure of probabilistic prediction error.

SHAP analysis provided an additional layer of interpretability by identifying the variables that contributed most strongly to model predictions. The prominence of total fever duration and IAI suggests that the model was not driven solely by isolated laboratory abnormalities, but by a clinically coherent pattern of persistent fever and systemic inflammatory injury. At the same time, SHAP values represent feature contributions to model prediction within this dataset. They should not be interpreted as causal effects or as evidence that the identified variables directly mediate severe disease, inflammasome activation, or disease progression.

The findings have potential implications for clinical risk stratification, but they should be considered exploratory. In routine pediatric practice, direct inflammasome assays are rarely available, whereas fever history and basic laboratory tests are widely accessible. A model incorporating these variables could potentially help identify children who require closer monitoring, earlier escalation of supportive care, or enrollment into future mechanism-guided studies. In the present revision, we further performed an exploratory single-center temporal validation using a small post-2019 cohort. The frozen random forest model retained moderate discriminative ability in this cohort, with an AUC of 0.840 and a Brier score of 0.173. This finding provides preliminary support for temporal robustness, but the cohort was small and derived from the same institution; therefore, it should not be interpreted as definitive external validation.

More importantly, the inflammatory injury-high phenotype may help select patients for prospective biomarker studies measuring IL-1β, IL-18, caspase-1 activity, NLRP3 expression, and gasdermin D cleavage. If validated, such a phenotype could provide a clinically practical bridge between bedside severity assessment and inflammasome-oriented translational research. At this stage, however, the IAI should be regarded as a research-derived composite index for phenotype characterization and model interpretation, rather than a validated bedside scoring system. Prospective multicenter studies are required before the model or IAI can be considered for clinical decision-making.

Several limitations should be acknowledged. First, this was a retrospective, single-center study with a modest sample size, which limits generalizability and increases the risk of optimistic performance estimates. Although repeated stratified cross-validation and out-of-fold prediction were used, independent multicenter external validation was not available (Collins et al., 2024). We added a small post-2019 single-center temporal validation cohort in the revised analysis, and the frozen random forest model showed moderate discrimination in this cohort. However, this cohort included only 39 patients and was derived from the same institution. Therefore, it should be interpreted as exploratory temporal validation rather than definitive external validation. The reported model performance should be interpreted as internal evaluation rather than evidence of clinical deployment readiness.

Second, fever duration reflected the total febrile course rather than a variable strictly available at initial presentation. Accordingly, the present model should be interpreted as a clinical severity-discrimination and phenotype-discovery framework rather than a purely admission-based prediction tool. Future studies should validate an admission-only model using variables strictly available at initial presentation.

Third, some variables, particularly albumin and CK-MB, had substantial missingness. Median imputation was performed within the modeling pipeline, and a sensitivity analysis excluding these variables still showed meaningful discrimination. However, imputation cannot fully replace systematic prospective data collection. Future prospective studies should minimize missing data and standardize laboratory assessment.

Fourth, the IAI is a surrogate clinical index and does not directly measure inflammasome activation. Direct molecular validation using NLRP3, caspase-1, IL-1β, IL-18, and gasdermin D is essential. In addition, the current IAI cannot distinguish inflammasome-mediated inflammation from other inflammatory or tissue-injury pathways. Therefore, the term “inflammasome-associated” should be understood as a biologically motivated clinical inference rather than direct molecular evidence.

Fifth, adenovirus serotype and viral load information were not consistently available, preventing analysis of whether specific viral characteristics, such as HAdV-7 infection or higher viral burden, were associated with the inflammatory injury-high phenotype.

Sixth, the model focused on severity classification rather than longitudinal outcomes such as mechanical ventilation, intensive care admission, bronchiolitis obliterans, mortality, or long-term pulmonary dysfunction. Other clinically relevant outcomes, including pediatric intensive care unit admission, duration of oxygen therapy, complications, and long-term respiratory sequelae, were not systematically available in this retrospective dataset. Therefore, the inflammatory injury-high phenotype should be interpreted as a severity-enriched inflammatory injury subgroup rather than a validated prognostic subgroup.

Finally, because the IAI was derived from several component variables that were also included in the modeling framework, feature-importance estimates should be interpreted cautiously. Future studies should compare original-variable-only, IAI-only, and non-overlapping IAI-based feature sets.

Future research should prospectively validate this framework in larger multicenter pediatric cohorts. Such studies should use strictly admission-based variables, incorporate adenovirus serotyping and viral load, and collect direct inflammasome-related biomarkers. Longitudinal sampling would help clarify whether the IAI reflects early inflammatory risk, ongoing disease activity, or downstream consequences of established severe disease. In addition, calibration analysis, decision-curve analysis, and external validation are needed before any model-derived risk score can be considered for clinical use. Ultimately, a clinically useful framework should combine accurate severity discrimination, transparent model interpretation, and biological validation of clinically inferred inflammasome-associated inflammatory injury. Future prospective studies should also evaluate whether the inflammatory injury-high phenotype is associated with clinically meaningful outcomes, including pediatric intensive care unit admission, mechanical ventilation, duration of oxygen therapy, complications, mortality, and long-term pulmonary sequelae.

## 5 Conclusion

This study developed a retrospective interpretable machine-learning framework for clinical severity discrimination in severe adenovirus pneumonia in children using routinely available clinical and laboratory variables. Total fever duration and the surrogate Inflammasome-associated Inflammatory Injury Index were the leading contributors to model prediction, and unsupervised clustering identified an inflammatory injury-high phenotype that was strongly enriched for severe disease. Exploratory single-center temporal validation in a small post-2019 cohort suggested that the frozen random forest model retained moderate discriminative ability, but this finding requires confirmation in larger independent cohorts.

These findings suggest that severe pediatric adenovirus pneumonia may involve a clinically detectable systemic inflammatory injury pattern that is biologically compatible with inflammasome-related pathophysiology. However, the IAI is a surrogate clinical index rather than a direct molecular measurement of inflammasome activation. Prospective multicenter studies incorporating adenovirus serotyping, viral load assessment, and direct inflammasome-related biomarkers, including NLRP3, caspase-1, IL-1β, IL-18, and gasdermin D, are required to validate this framework and clarify its translational significance.

## Data Availability

The raw data supporting the conclusions of this article will be made available by the authors, without undue reservation.
