# Supplementary material for: Interpretable machine learning identifies a clinically inferred inflammasome-associated inflammatory injury phenotype in children with adenovirus pneumonia
Source: Front Cell Infect Microbiol. 2026 Jul 17;16:1902566. doi: 10.3389/fcimb.2026.1902566 (PMC13424107; doi:10.3389/fcimb.2026.1902566)
Supplement: Supplementary file 1 [file Table1.docx]

**Supplementary Table S1. Exploratory single-center temporal validation performance of the frozen supervised machine-learning models.**

| **Model** | **AUC** | **AUC 95% CI** | **PR-AUC** | **Accuracy** | **Precision** | **Sensitivity** | **Specificity** | **F1-score** | **Brier score** |
| --- | --- | --- | --- | --- | --- | --- | --- | --- | --- |
| **Random forest** | **0.840** | **0.695–0.954** | **0.831** | **0.744** | **0.667** | **0.750** | **0.739** | **0.706** | **0.173** |
| **SVM-RBF** | **0.796** | **0.631–0.926** | **0.779** | **0.744** | **0.714** | **0.625** | **0.826** | **0.667** | **0.188** |
| **Logistic regression** | **0.772** | **0.596–0.915** | **0.767** | **0.769** | **0.818** | **0.562** | **0.913** | **0.667** | **0.190** |

The exploratory temporal validation cohort included 39 post-2019 cases from the same institution, comprising 23 mild and 16 severe cases. The frozen models developed from the original cohort were directly applied without retraining, feature reselection, threshold adjustment, or recalculation of preprocessing parameters. AUC = area under the receiver-operating-characteristic curve; PR-AUC = area under the precision-recall curve; SVM-RBF = support vector machine with radial basis function kernel.
