# Supplementary material for: Interpretable machine learning identifies a clinically inferred inflammasome-associated inflammatory injury phenotype in children with adenovirus pneumonia
Source: Front Cell Infect Microbiol. 2026 Jul 17;16:1902566. doi: 10.3389/fcimb.2026.1902566 (PMC13424107; doi:10.3389/fcimb.2026.1902566)
Supplement: Supplementary file 2 [file DataSheet1.docx]

**Supplementary Figure S1. ROC curves for exploratory single-center temporal validation.**


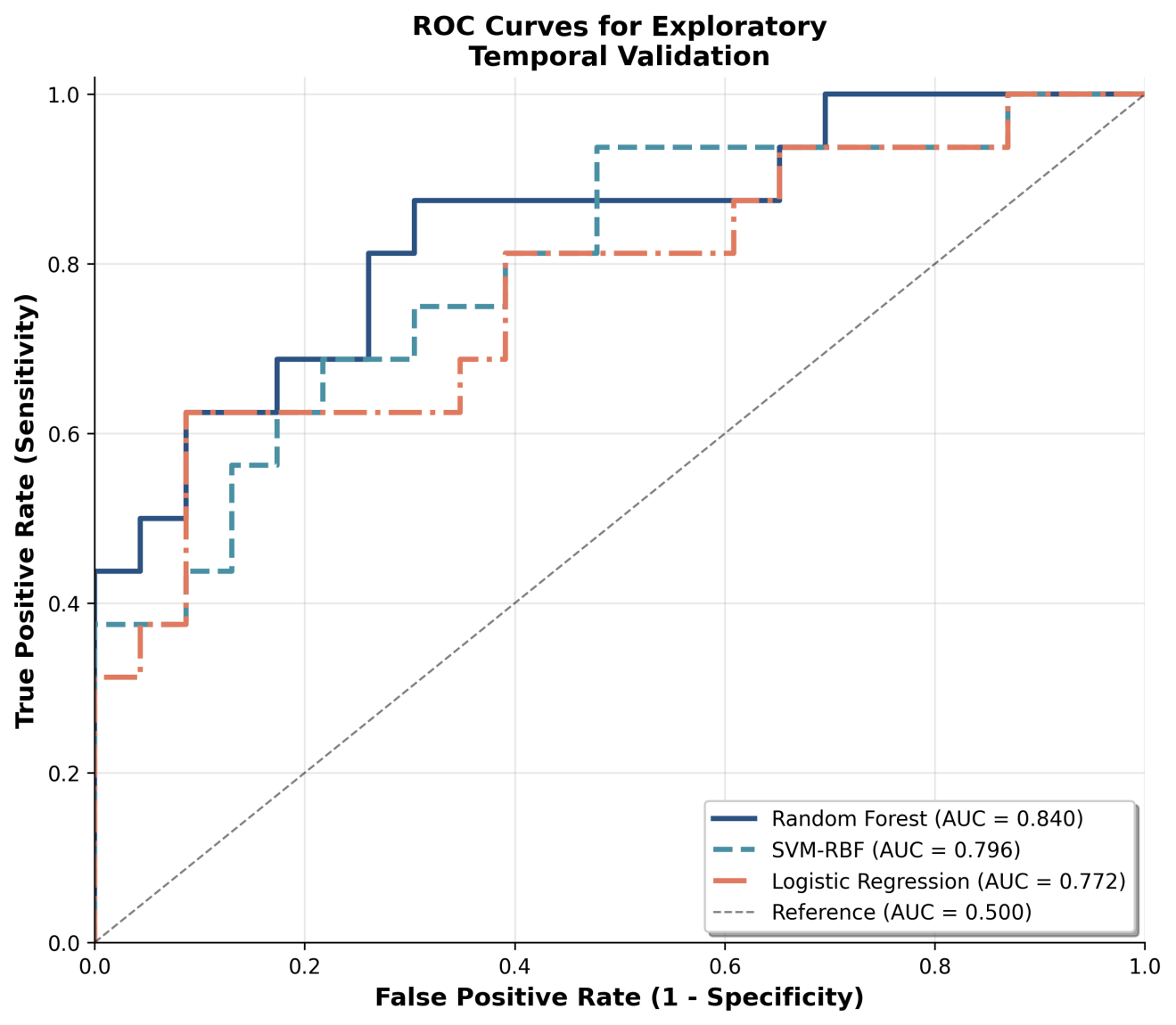


ROC curves were generated by applying the frozen models to the post-2019 exploratory temporal validation cohort. This cohort included 39 children with adenovirus pneumonia, comprising 23 mild and 16 severe cases. The frozen models were applied without retraining, feature reselection, threshold adjustment, or recalculation of preprocessing parameters. The random forest model showed the highest discrimination, with an AUC of 0.840. These results should be interpreted as exploratory temporal validation rather than definitive external validation. ROC = receiver-operating-characteristic; AUC = area under the receiver-operating-characteristic curve.

**Supplementary Figure S2. Descriptive calibration plot for the frozen random forest model in the exploratory temporal validation cohort.**

**
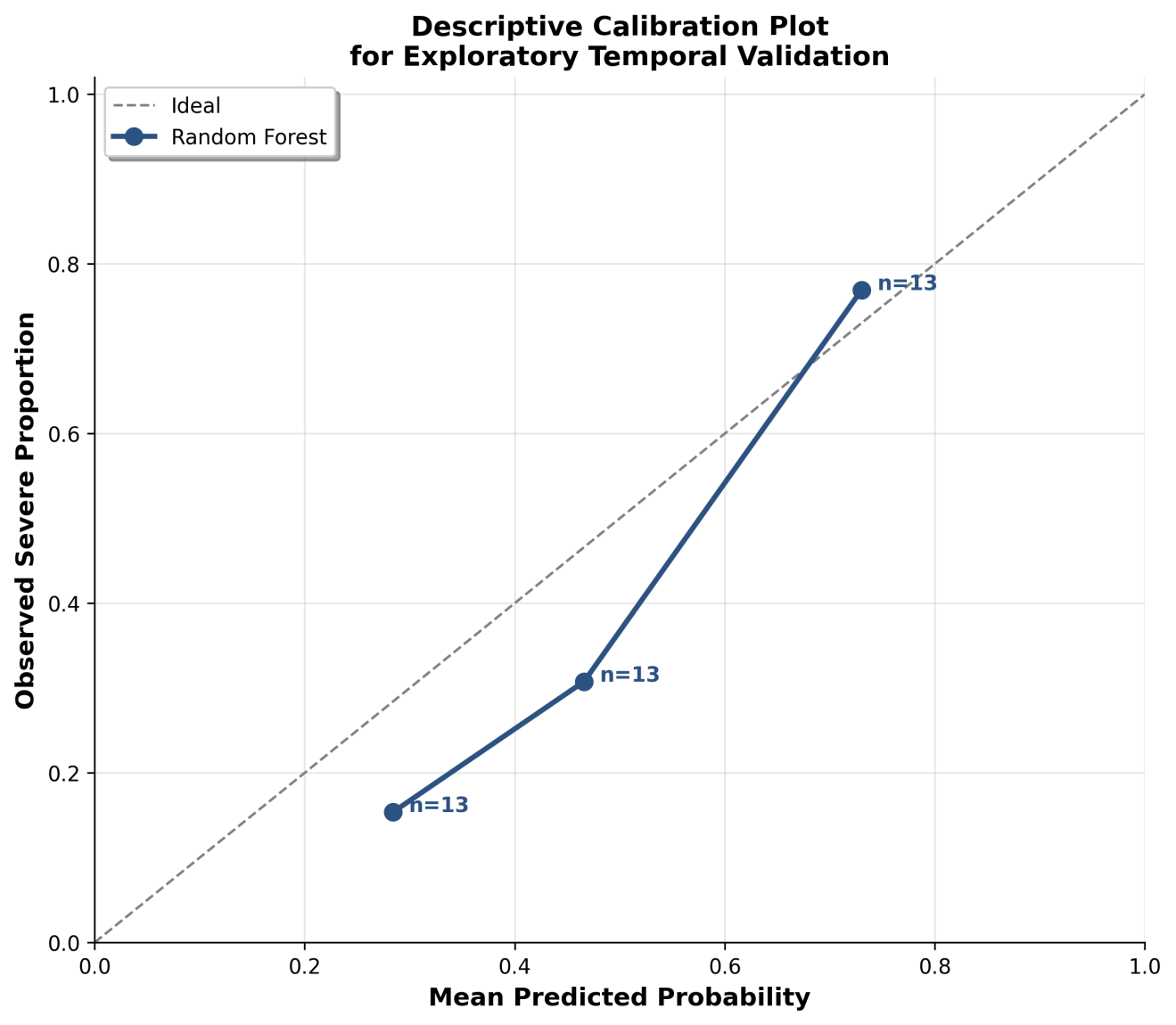
**

The calibration plot shows the relationship between predicted probabilities from the frozen random forest model and observed severe-case proportions in the exploratory temporal validation cohort. Because this cohort was small, the calibration plot was generated for descriptive visualization only. The Brier score of the random forest model in this cohort was 0.173. These results do not represent definitive calibration validation and require confirmation in larger prospective multicenter cohorts.
